# Editorial: Cell Adhesion and Migration in the Development of Multicellular Organisms

**DOI:** 10.3389/fcell.2018.00142

**Published:** 2018-10-22

**Authors:** Takaaki Matsui

**Affiliations:** Gene Regulation Research, Division of Biological Science, Graduate School of Science and Technology, Nara Institute of Science and Technology, Nara, Japan

**Keywords:** cell migration, collective cell migration, development, multicellular organisms, cell adhesion

Cells express many adhesion molecules, cytoskeletons, extracellular matrices, and signal activators/mediators, that are involved in cell adhesion and migration. To generate well-organized tissues during the development of multicellular organisms, expression and/or function of these molecules need to be controlled appropriately in a spatiotemporal manner. In addition, individual regulation and orchestration of cell adhesion and migration are also important. In this research topic, we aimed to understand how cell adhesion and migration are regulated and orchestrated during development and how cell adhesion and migration affect tissue formation during development, and to that end, have put together a collection of articles related to this research area.

On the topic of adhesion molecules, Watanabe produced a review that focuses on the role of gap junction proteins in skin pattern formation and body shape determination, while a review by Togashi touched on the differential and cooperative roles of cadheins and nectins in sensory organ patterning. In another review, Sanghvi-Shah and Weber elaborated on the role of the intermediate filaments in mechanotransduction signaling, and proposed that intermediate filaments act as a centerpiece between mechanical stimuli and directional cell migration.

And along the same line, Hirota and Nakajima; Matsubara et al. also produced interesting reports that focus on signaling mechanisms of cell migration and adhesion in cerebrum and limb, respectively. On the other hand, mechanism of cell-chirality-driven tissue rotation, which is required for proper left-right patterning, is elegantly discussed by Inaki et al. Equally intriguing is the report by Hiraiwa et al. that uses numerical simulation study to show the importance of wave-like propagation of junctional remodeling to collective cell migration.

Using an approach that combined live imaging and computational biology, Sakane et al. and Tsuboi et al. further succeeded in quantifying the fascinating dancing style of collective cell migration and to infer cell mechanics within heterogeneous epithelial tissue, respectively. And in an another advanced technical approach, Nagasaka et al. was able to measure cellular stiffness at the tissue- and single-cell-levels by using an atomic force microscope (AFM) and showed that tissue-level stiffness is determined not only by mechanical properties of single cells but also by cellular densification, and affects nuclear/somal movements of neuroepithelial cells in cerebrum development. These curated articles showed the variety of opinions that are currently forming in regards to regulation of cell adhesion and migration in various organisms including mouse, ferret, fish, and fly. Nevertheless, they shared the same core points emphasizing that spatial and temporal regulations of expression and/or dynamics of adhesion molecules and cytoskeletons through signal transduction and mechanotransduction are required for proper regulation of cell adhesion and migration, and that mechanical properties of cells and/or mechanical interactions among cells affect cell adhesion and migration during development. In addition, this research topic suggests that interdisciplinary approaches of developmental biology, mathematics, and/or physics would be an excellent system to understand how cell adhesion and migration are regulated and orchestrated during development. By assembling these articles together under this research topic, we hope that it would deepen the current understanding of cell adhesion and migration during the development of multicellular organisms, and serve as the basis for the future works geared to advance this research area.

## Author contributions

The author confirms being the sole contributor of this work and has approved it for publication.

### Conflict of interest statement

The author declares that the research was conducted in the absence of any commercial or financial relationships that could be construed as a potential conflict of interest.

